# Humans as Animal Sentinels for Forecasting Asthma Events: Helping Health Services Become More Responsive

**DOI:** 10.1371/journal.pone.0047823

**Published:** 2012-10-31

**Authors:** Ireneous N. Soyiri, Daniel D. Reidpath

**Affiliations:** 1 SEACO, Monash University, Bandar Sunway, Selangor, Malaysia; 2 School of Public Health, University of Ghana, Accra, Ghana; University of Montreal, Canada

## Abstract

The concept of forecasting asthma using humans as animal sentinels is uncommon. This study explores the plausibility of predicting future asthma daily admissions using retrospective data in London (2005–2006). Negative binomial regressions were used in modeling; allowing the non-contiguous autoregressive components. Selected lags were based on partial autocorrelation function (PACF) plot with a maximum lag of 7 days. The model was contrasted with naïve historical and seasonal models. All models were cross validated. Mean daily asthma admission in 2005 was 27.9 and in 2006 it was 28.9. The lags 1, 2, 3, 6 and 7 were independently associated with daily asthma admissions based on their PACF plots. The lag model prediction of peak admissions were often slightly out of synchronization with the actual data, but the days of greater admissions were better matched than the days of lower admissions. A further investigation across various populations is necessary.

## Introduction

Asthma is a chronic respiratory illness of immense global proportions, and it affects over 300 million people. Recent reviews have reaffirmed the highly heterogeneous nature of the disease, which is influenced by complex genetic and environmental effects as well as an evolving knowledge-base of its key determinants [1]. Many of these reviews comprehensively addressed the key factors which contribute to the manifestation and progression of asthma in individuals and lab based experiments [2], [3], [4]. There was however less content on the forecasting of asthma events for the purposes of providing early warning systems to help manage the condition in larger populations. Meanwhile, an approach to develop a forecast for respiratory conditions that are dependent on environmental exposures (e.g. asthma), which is yet to be reported in the literature, is the use of humans as animal sentinels to forecast asthma.

The classical animal sentinel is the canary in the coal mine. Coal miners would carry a caged canary with them into mines knowing that the birds were more sensitive to the toxic gases found in the coal seams than were the miners [5], [6], [7]. If the canary died then the humans had early warning about the presence of toxic gases and could evacuate the mine.

Since those times animal sentinels have been widely used for monitoring changes in environmental exposures [3], [8], [9], [10]. Although it is not usually discussed in these terms, there is also a potential for humans to act as animal sentinels for environmental exposures for other humans. The use of syndromic surveillance to detect non-infectious bioterrorism is an example of this [11], [12]. Unlike animal sentinels, however, where specific identifiable animals are followed up over time, human sentinel surveillance follows fluctuations in health events over entire populations. The logic is that people who are more sensitive to environmental exposures or (because of geographic location) people who experience earlier exposure will present in hospital records sooner than the less sensitive. As the dose of an environmental exposure increases (or diffuses across the population), so more people will experience health events. Thus, temporal fluctuations in the numbers patients presenting to hospitals will be, in part, attributable to fluctuations in environmental exposure.

There is the potential to utilize human sentinels for predicting more routine variations in disease events to inform health service provision. For example, in the case of asthma events, those people with more sensitive lungs are likely to respond more quickly to changes in environmental exposure than those people with less sensitive lungs. In effect, the sensitive lung is “the canary in the coal mine” for the less sensitive lung. Without having to measure any particular environmental trigger or determine the causal relationships between environmental exposures and asthma events, the potential exists to use the frequency of asthma events today to predict the frequency of asthma events in the future and feed this into decision making about health services provision.

Previous studies have looked at the forecasting of asthma events, but have tended to focus on relationships between the environmental exposures which are known to trigger asthma events, such as weather conditions or Ozone and PM10 levels, as well as the extent to which these can be used to forecast asthma [13]–[18]. Other related studies, such as the recent study by Eisner and colleagues on the use of an assessment tool for measuring the “severity of asthma score” and using it to predicts clinical outcomes in patients with moderate to severe persistent asthma, have demonstrated the predictability of adverse clinical outcomes in specific group of patients (i.e. moderate to severe asthma) [19]. In contrast, it is the aim of the present study to ignore the specifics of any environmental exposure or demographic factor(s), and focus exclusively on the possibility of using sentinel humans living within the community to forecast asthma events. If asthma sufferers can be used as sentinels for other asthma sufferers, the possibility exists that by monitoring changes in the number of asthma events, health services would be able to respond more efficiently to the future demands. As a result individual asthma sufferers could be alerted to their personal increased risk. The plausibility however needs to be established first before the potential value to health issues can be explored.

The objective of this study was to examine the relative value of autoregressive models to forecast asthma admissions using data for two years of hospital admissions for asthma from London (2005–2006). Because the interest is forecasting performance, and there is no sense in which one can suggest that the lagged count of asthma admissions from some days ago caused the asthma admissions of today, reporting the parameter estimates for particular lags are likely to be of little value, or misleading [20]. We focus, therefore on the more relevant predictive performance of the models

## Methods

This study involved the development of an asthma forecasting model based on a secondary analysis of hospital administrative data from London, England. The data covered 20,794 hospital admissions that occurred within the perimeter formed by the M25 Motorway (surrounding London) where the admissions had a primary diagnosis of asthma.

### Data

Data were sourced from the nationally recorded Hospital Episode Statistics (HES) maintained by the National Health Service, England [21]. Asthma admissions were defined as any hospital admission with a primary diagnosis of asthma; i.e., an International Classification of Diseases (ICD-) 10 code of J45. The data covered all days between January 1st, 2005 and December 31st, 2006 with no missing data.

The outcome variable for the study was the daily count of admissions for asthma. The predictor variables were selected lags of previous days' admissions. The selection of lags is explained in the following section (Data Analysis). The data were divided into two annual sets: a model development data set from the 2005 admissions data and a cross validation data set from 2006 admissions data.

Based on the aggregate, anonymous and administrative nature of the data, an exemption from ethical review for the secondary analysis was obtained from the Monash University Human Research Ethics Committee (Number: 2011001092).

### Data Analysis

The analysis of the data relied on a comparison of forecasting models of asthma daily admissions in which 2005 hospitals admissions data was used in the development of three negative binomial regression models, and 2006 data were used for cross-validation. The three models were:

A mean daily admissions (historical model). This model was a null model that included no predictor variables.

A seasonal model: The seasonal model included three dummy predictor variables to model the effects of the four seasons. Season was dummy coded, in keeping with earlier work using these data, because this fitted the data better than a smoothed seasonal model.

An autoregressive (lags) models: A lag represents the admissions count from a previous day. Thus a 1 day lag represents the admissions count from the day before the day being modelled, and a two day lag represents the admissions count from two days prior to the day being modelled. The lags model included the non-contiguous lagged data from the days prior to the modeled day as predictor variables. The lags were informed by a partial autocorrelation function (PACF) plot with a maximum lag of 7 days.

Negative binomial regression was chosen for the modelling because the asthma daily admissions counts were known to have issues with over dispersion, [22]–[27]. Following Hilbe, [28] the probability model can be conceptualised in the following way. *P* is the probability function of the negative binomial distribution:
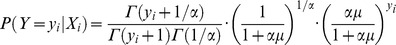
Where: *y_i_* represents the number of admissions; *μ* = exp(*X_i_*β); β is the vector of coefficients; *X_i_* is the vector of predictor variables (in this case “1” for the historical model, the dummy variables of three seasons for the seasonal model, and the admissions counts for the lagged days 1, 2, 3, 6 and 7 for the lags model); *α* is the overdispersion parameter; and Γ is the gamma function The predictor variable parameters (β) were estimated via maximum likelihood estimation.

A positive coefficient in the regression output indicates that a factor will increase the number of daily asthma admissions relative to its reference category and conversely a negative coefficient will decrease the number of daily asthma admissions relative to its reference category. The exponent of the coefficient can be interpreted, all other things being equal as the proportionate increase (for values greater than 1) or decrease (for values between 0 and 1) of number of daily asthma admissions associated with a one unit increase in the predictor variable [27], [28]. The predictor variable(s) herein refers to the functional form of the lag term(s) constituting the NBM. As stated in the objective of this study, this univariate model does not account for other plausible indicators of asthma (e.g. pollution) other than lagged asthma events. We acknowledge that, accounting for multivariable factors is beyond the scope of this paper, even though they may be viewed as potentially confounding risk factors that are also time dependant. Hence for our analyses, specific potential covariates were selected nonlinear lags of 0 to 7 days of asthma admissions from the training dataset (i.e. 2005 asthma daily admissions in London). To the best of our knowledge, there is no standard reference in current literature for lag selection for this kind of study, as it has not been carried out before. Hence our choice of this range of lags was to satisfy the biological plausibility of our hypothesis and also develop a tool which relies on a “short memory”. The selection of lag combinations for the models involved a computationally exhaustive process, selecting the best fit for all possible lags.

### Model Formulation

Three models were developed for comparison purposes, using the 2005 data. The first model was the mean daily admissions (historical) model. The final model utilized non-contiguous autoregressive lags. Season was *dummy* coded, in keeping with earlier work using these data that indicated a better fit than with a smoothed seasonal model.

1. Mean daily admissions (historical model): This model was defined by a function of the average daily asthma admissions in London in 2005;
2. Seasonal model: Then seasonal model was defined by four meteorological seasons, categorized as dummy variable;
3. Lags models: The lag model was defined by a function of combinations of the 0–7day lags which yielded the best predictive model. The model comprised a multivariable 1, 2, 3, 6 and 7 day lags.

### Error measures

Three measures of fit were used to evaluate modeled data for 2005 and the predictive forecast of the model on the cross-validation data from 2006. The measures of predictive performance were R-squared, root mean squared error (RMSE) and mean absolute scaled error (MASE) [29]. RMSE was included because it is well known and still popular in the literature although it has known problems [30]. R-squared, though flawed as a measure of predictive validity, [31] remains popular and was included purely for historical reasons. MASE is now regarded as one of the better measures of predictive validity, [32] but it requires a scaling factor against which to measure performance. The scaling factor was derived from the mean absolute error of the predictions based on the 2005 historical mean daily admissions. When interpreting the measures of error, it should be noted that with the exception of R-squared, smaller numbers indicate less error between the forecast and actual data. In contrast, larger R-squared values are indicative of a better fit between the forecast and actual data.

Analyses were conducted using the R (Version 2.14.1) statistical environment [33] and Stata (version 11.2) statistical package [34].

## Results

The mean daily asthma admission in 2005 was 27.9 and in 2006 it was 28.9. The plot of the PACF indicated lags 1, 2, 3, 6 and 7 were independently associated with daily asthma admissions. These plots lie within reasonable confidence bounds (i.e. 95% Confidence Interval). The negative binomial regression model was developed using these lags.

**Figure 1:**
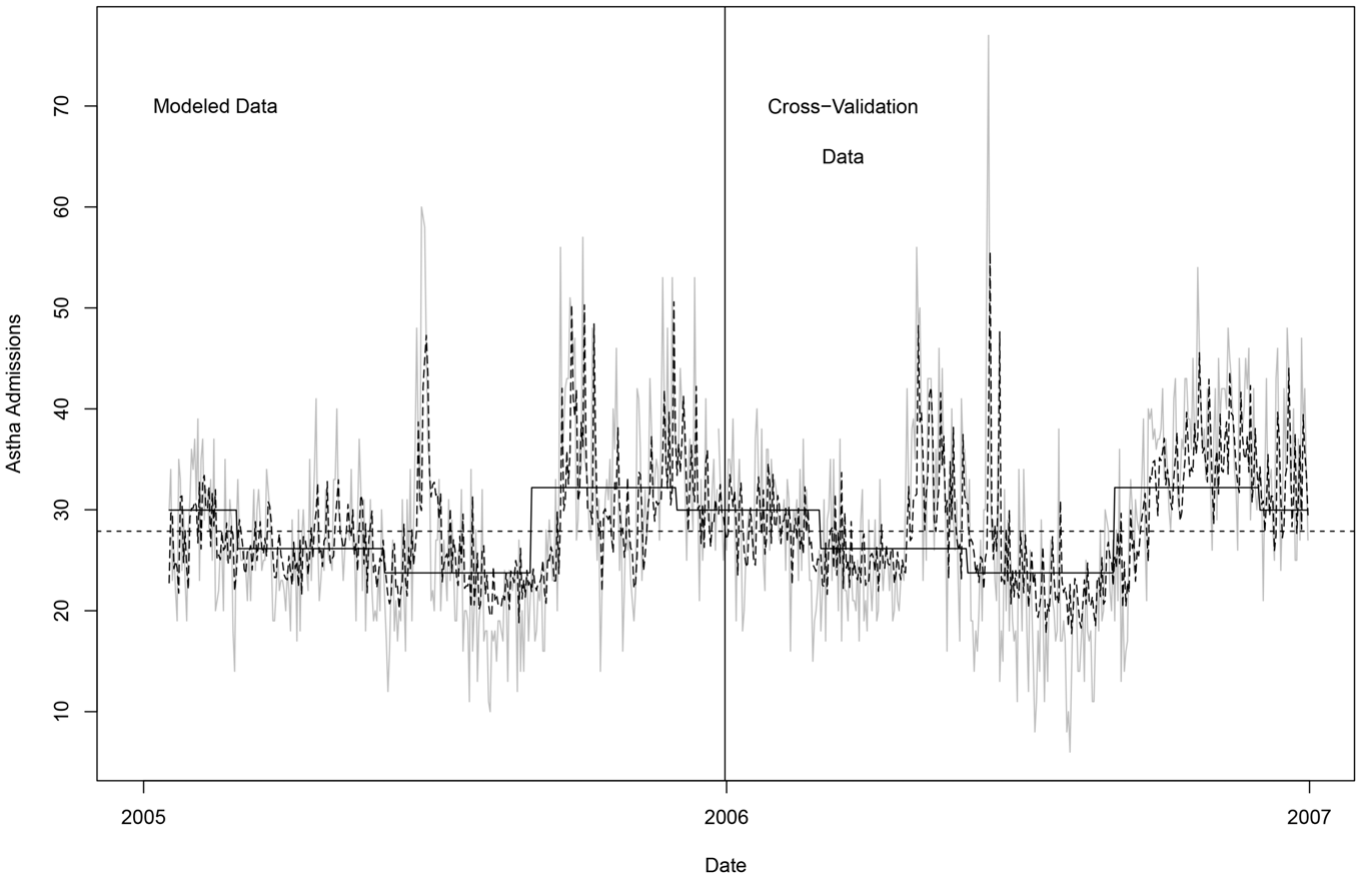
A plot of Asthma daily admissions in London (2005–2006). The grey line represents a plot of the actual asthma admissions data in London (2005–2006); The dashed black line shows the lag model of asthma daily admissions in London (2005–2006); The solid black line shows the seasonal model's plots; The straight dashed line represents the historical model; and The solid vertical line (1 January 2006) shows the division between the data on which the models were developed and the data on which the models were cross-validated.

Figure 1 shows a plot of the asthma admissions data (grey line), and the lag model (dashed black line), seasonal model (solid black line) and the historical model (straight dashed line). A solid vertical line (1 January 2006) shows the division between the data on which the models were developed and the data on which the models were cross-validated (i.e., the predictive forecasts were measured).

It appears from the figure that the lag model captures the daily variation in the admissions better than the seasonal model, which is certainly better than the historical model. Careful scrutiny of the figure however shows the peak admissions predicted by the lag model are often slightly out of synchronization with the actual data. It also appears that the days of greater admissions are somewhat better matched than the days of lower admissions.

Table 1 shows the measured fit of the lag model, the seasonal model and the historical model. The scaling factor for the MASE measure was derived from the historical model. As a consequence, the MASE for the historical model for 2005 is 1, and all comparisons of fit relate to the fit of the historical model.

**Table 1:** Measures of fit for the historical, seasonal, and lag models for asthma daily admissions in London, 2005 and 2006.

Error Measure	2005 (Model)	2006 (Forecast)
R^2^ Historical	*	*
R^2^ Seasonal	0.146	0.235
R^2^ Lag	0.366	0.376
RMSE Historical	8.75	9.65
RMSE Seasonal	8.09	8.55
RMSE Lag	6.97	7.57
MASE Historical	1.000	1.150
MASE Seasonal	0.887	0.977
MASE Lag	0.784	0.857

* R^2^ values cannot be computed for these models, because there is no variation in the predicted daily admissions.

## Discussion

Using human sentinels to forecast asthma events in large concentrated populations is uncommon. Previous studies on animal sentinels have tended to use mammals, which occupy shared environments and/or exposures with humans [10]. This study makes an important contribution by using retrospective asthma admission records in London to demonstrate the plausible hypothesis.

The idea of forecasting asthma using human sentinels was based on the probable observation that asthma sufferers with more sensitive lungs, all things being equal, would react more to environmental changes or to the precursors of asthma exacerbations than their less sensitive counterparts. Where others have considered lagged effects of pollutants on asthma, and sometimes included autoregressive components in their analysis, these have not been used for forecasting [13]–[18]. Where research has been conducted on forecasting of asthma (and other respiratory conditions), this has not considered autoreggressive predictors [19].

There is no consensus on the approach to developing health forecasting models. There is also no agreed scale in determining what constitutes a good health forecast model, but for the fact that such a model predicts well. The modeling approach described in this study is quite flexible because it provided an opportunity to choose the most suitable predictors and guarding against over fitting of the model by limiting the range of lags (covariates) to be selected.

Partial autocorrelation function plots (and other model diagnostic tools like Plot of time series residuals, Normal quantile plot and Autocorrelation function) have been found to be useful guides in selecting covariates for modeling and prediction [35], [36]. A key advantage of this model building approach is that it combines fast input selection with accurate but computationally demanding non-linear predictions [37]. Additionally, the complexity of the input variable selection process makes the approach viable for large scale population health challenges. Ultimately, it still provides a wide range of potential models for the best forecast model to be selected based on the chosen measures of fit and cross validation.

### Forecasting and error measures

There is little difference in the R^2^ for the lag model in 2005 or 2006. Both measures account for a little over 35% of the variation in asthma daily admissions. The seasonal model, surprisingly, accounts for a greater proportion of the variation of asthma daily admissions in the cross validation period.

The RMSE statistics show that the lag model consistently out performs the seasonal model, which in turn consistently out performs the historical model. For the modeled data (2005), the seasonal model has an RMSE around 8% smaller than the historical model and the lag model has and RMSE about 21% smaller than the historical model. In the cross validation period (2006), the forecast predictions of all the models are (as expected) worse than they were for the modeled data. The rank order however remains unchanged, with the lag model out performing either of the other models. With respect to MASE, the seasonal models performance is around 15% lower than the performance of the historical model, and the lag model is around 25% lower than the performance of the historical model

The preference of MASE over RMSE and R^2^ as an error measure for forecasting has also been discussed by previous authors [29], [32]. The MASE statistics are more easily interpreted, and potentially the most reliable and informative measure of accuracy in forecasting [29]. It is widely recommended for comparing forecast accuracy across series on different scales, because it is a scaled error measure. Hyndman and Koehler, (2006) have also reported that MASE provides the most reliable approach because of its meaningful scale, which is widely applicable and less prone to “degeneracy” problems [32]. Furthermore, MASE shows smaller variations, even with small samples, than other measures in the same category and is also known to be less sensitive to outliers [32], [38]. The use of MASE as a standard measure of accuracy may therefore enhance the utility of our lagged models in comparing the predictions of asthma daily admissions across various populations.

A comparison of the forecast models within the model development sample (i.e. *Modeled* data), and equally within the test sample (i.e. *Cross-Validation* data) shows various degrees of contrast between the three models we have presented. The observed contrasts between models that are within the same sample frame are useful for benchmarking and selecting the best model to be used in future predictions. These differences are attributed to the constituents (or covariates) of each specific model. On the other hand, it is expected that there are marked differences between the model parameters of the *Modeled* and *Cross-Validation* datasets because, their distributions vary as well. One important issue worth noting and also further investigation is the fact that the lag model predicts asthma daily admissions better during peak periods than moments of low admissions. Further analysis on the relationship between prediction and variations in admission rates is also recommended.

### Limitations of study

A major limitation to this approach to forecasting asthma is the data sources and reliability. In this study we anticipated one major limitation could be from the inherent inaccuracies (reliability) of the original data/records. Generally it is assumed that everyone experiencing an asthma exacerbation would be recorded in the database, but conversely, some individuals may seek alternative care and hence go unnoticed. Also, issues of misdiagnoses could be a contributory factor to the data limitations.

In some regards, our choice of treating all cases as unique, including repeat admission cases in the dataset, may be seen as a limitation because of the unique characteristics of such individuals. Nevertheless, from a service provider's perspective, it may make no significant difference.

### Implications of the study

This study aims at demonstrating a novel approach to developing an early warning system, which could then be used by health service providers. We however, do not anticipate that results of this current study would be used without circumspect, but hope that the procedure should be validated with larger population datasets and preferably across various populations. If this is done, we can be hopeful that health service providers, individual asthma sufferers and their care providers can be duly informed of when to expect peak and low asthma exacerbations. Such information, which comes as a guide, can enhance health policy decisions and resource allocation, health promotion via anticipatory care/management strategies for asthma and overall minimize the disease burden of the condition.

### Conclusions

Uncertainty and chance is an inexorable element of any forecasting system or approach. Nonetheless this study highlights that, detailed and comprehensive retrospective records of asthma daily events can be used in forecasting future events. The study demonstrates that Lag models predict peak asthma admissions better than lower admissions.

All the three error measures (R^2^, RMSE and MASE) were consistent in both the modeled data and cross-validation datasets.

The knowledge of the underlying relationships between asthma daily admissions and related lag events that precede the former has provided an underpinning prediction approach of future events. This approach to forecasting does not include other potential predictors that may be known as confounders, and thus minimizes the potential error in predictions associated with their measurement errors. However, important questions that remain unanswered include how such a proposed forecasting model will perform in different settings for different populations, and the precise mechanisms that will be most suitable for modifying the predictors of the respective population data.
